# Microemulsion Delivery Systems with Low Surfactant Concentrations: Optimization of Structure and Properties by Glycol Cosurfactants

**DOI:** 10.1021/acs.molpharmaceut.2c00599

**Published:** 2022-11-10

**Authors:** Patrycja Szumała, Jolanta Kaplińska, Balbina Makurat-Kasprolewicz, Szymon Mania

**Affiliations:** † Department of Colloid and Lipid Science, Faculty of Chemistry, Gdańsk University of Technology, Narutowicza Street 11/12, 80-233 Gdańsk, Poland; ‡ Faculty of Mechanical Engineering and Ship Technology, Gdansk University of Technology, Narutowicza Street 11/12, 80-233 Gdansk, Poland; § Department of Chemistry, Technology and Biotechnology of Food, Gdansk University of Technology, Narutowicza Street 11/12, 80-233 Gdansk, Poland

**Keywords:** microemulsion, optimization, propylene glycol, pentylene glycol, low surfactant content

## Abstract

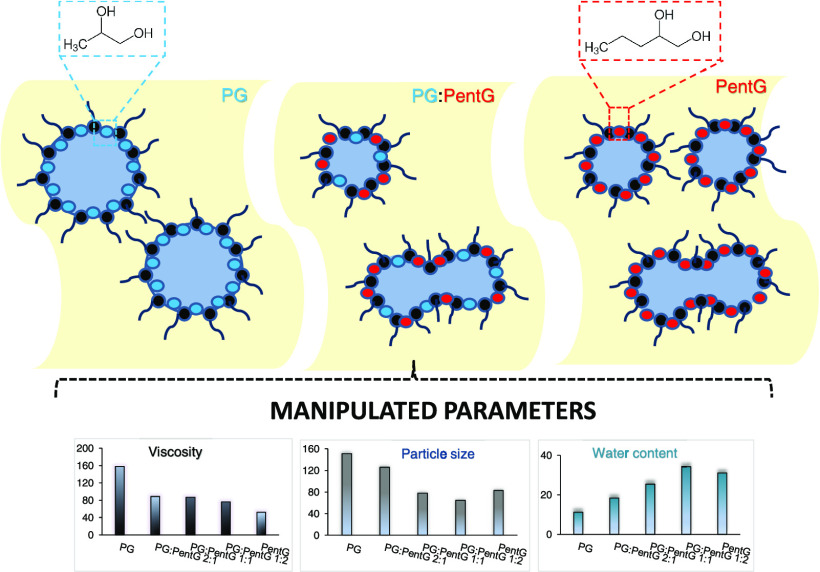

Extensive use of microemulsions as delivery systems raises interest in the safe ingredients that can form such systems. Here, we assessed the use of two glycols, i.e., propylene glycol and pentylene glycol, and their mixtures to manipulate the properties and structure of microemulsions. Obtained systems with glycols were extensively characterized in terms of capacity to incorporate water phase, droplet size, polydispersity, structure type, and rheological and thermal properties. The results of these studies indicate that the composition, structure, and viscosity of the microemulsions can be changed by appropriate quantification of glycols. It has been shown that the type of glycol used and its amount may favor or worsen the formation of microemulsions with the selected oils. In addition, a properly selected composition of oils and glycols resulted in the formation of microemulsions with a reduced content of surfactants and consequently improved the safety of using microemulsions as delivery systems.

## 1 Introduction

In the field of delivery systems, microemulsions (MEs) are one of the most studied colloids.^1−3^ These systems are composed of the water and oil phases as well as stabilizers, i.e., surfactants and, most often, cosurfactants. Their characteristic feature is transparency, caused by the particle size of the internal phase in the range of approx. 10–150 nm. Achieving the nanometric size of the droplets requires the use of an appropriate composition of components that ensures very low interfacial tension and spontaneous formation of the system. MEs are distinguished by their high ability to solubilize hydrophilic and hydrophobic components and thermodynamic stability.^4,5^

Cosmetic and pharmaceutical MEs require the use of safe ingredients that will provide the right conditions for the formation of such systems but will not cause a negative reaction in the body. The irritant potential can be caused mainly by stabilizing agents; therefore, it is recommended to use a mixture of surfactants or mixtures of surfactants with cosurfactants to lower their concentration in the system.^6−8^ Cosurfactants are generally short- and medium-chain alcohols, and polyols. In addition to stabilizing properties and reducing the concentration of surfactants, cosurfactants are used to increase the solubilizing capacity and to extend the ME existence region on the phase diagram.^9,10^ However, aliphatic n-alcohols with a carbon chain length of 3–8 have been ranked as strong irritants while ethanol has been considered as a moderate irritant.^11,12^ In the case of ethanol, special manufacturing conditions are also required to use this solvent in cosmetic products.

In our previous works, we obtained transdermal MEs that contained ethanol.^13,14^ However currently, more and more attention is paid to the possible adverse effects of this ingredient and difficulties with its use. Thus, the main goal of this work was to replace ethanol with glycol or a mixture of glycols in ME production. We selected two glycols, i.e., propylene glycol and pentylene glycol (PentG), because they have the ability to enhance the penetration of selected active substances into and across the skin, and are safe in cosmetic and pharmaceutical formulations.^15−17^ The other ME ingredients such as surfactants and oil phase were taken from our previously developed recipe.^13,14^ Both glycols have already been used to form MEs in other studies. However, to our knowledge, their use to control the formation, composition, and properties of MEs has never been analyzed before. Our research focused on determining the characteristic properties of MEs (structure, composition, rheological properties, and thermal behavior of various states of water) with the use of pure glycols and their three mixtures. In addition, we assumed designing MEs with a high content of dispersed aqueous phase (enables the introduction of significant amounts of active substances enclosed in the ME delivery system) and a reduced concentration of surfactants to overcome the major disadvantage (potential irritation caused by high concentration of surfactants) and increase the commercial use of MEs in the market.

## 2 Experimental Section

### 2.1 Materials

Surfactants: Span 80 (sorbitan monooleate, S80) and Tween 80 (polyoxyethylene sorbitan monooleate, T80) were purchased from Croda Poland. Cosurfactants: propylene glycol (99.5%, PG) and pentylene glycol (96%, 1,2-pentanediol, PentG) were purchased from POCH (Poland) and Sigma-Aldrich (Poland), respectively. Isopropyl myristate (IPM, Sigma-Aldrich, Poland), paraffin oil (Ondina 934, Techmasz, Poland), and medium-chain triglyceride (MCT, caprylic/capric triglyceride, Karlshamn, Sweden) were used as oil phases. The water used for the preparation of the MEs and solutions was redistilled water (κ = 0.06 μS/cm).

### 2.2 Preparation of Microemulsions

MEs with isopropyl myristate (IPM) as the oil phase, surfactants, i.e., S80:T80 (2:1 wt), and propylene glycol (PG) or pentylene glycol (PentG), or their mixtures, as cosurfactants were prepared. Water was added dropwise to the oil/surfactants + cosurfactant (constant ratio 2:1 wt of surfactants to cosurfactant) mixture with continuous stirring using a mechanical stirrer (300 rpm).^13,14^ The volume of water that caused the first turbidity in the system was noted. Then, the titration method was repeated until the last drop before turbidity. Each sample was further allowed to equilibrate at room temperature for at least 24 h before evaluation. To determine the quantitative composition of the obtained MEs, pseudoternary phase diagrams were constructed. For each phase diagram, the weight ratios of oil/surfactants + cosurfactant varied from 10:90 to 90:10. MEs were identified as the region where clear and transparent formulations were obtained upon visual inspection. Each sample was analyzed in triplicate, and the data were presented as the arithmetic mean.

### 2.3 Electrical Conductivity

During the emulsification, the electrical conductivity (TetraCon 325 conductivity cell, WTW, Germany) of MEs was continuously measured as water was added to determine the type and microstructural transition of the system. Samples were prepared with an electrolyte solution (NaCl 10^–2^ M) instead of pure water. Experiments were conducted in triplicate, and the average of the measurement data was used.

### 2.4 Particle Size

The average droplet size and dispersity (polydispersity index, PDI) of MEs were determined using a noninvasive backscatter light method (NIBS). A Zetasizer Nano ZS (Malvern Instruments, Malvern, United Kingdom) equipped with a helium–neon laser diffraction operating at 633 nm was employed. Measurements were performed at a given temperature of microemulsions formation (25 °C) in triplicate.

### 2.5 Rheological Properties

Rheology measurements were performed using a Brookfield Viscometer DV2T HA (LaboPlus, Poland). The cone/plate sets with a CPA-41Z spindle were used. The shear stress, shear rate, and viscosity of ME samples were measured at 20 ± 0.1 °C. The viscosity curves were used to characterize the rheological properties of MEs. Measurements were made in triplicate.

### 2.6 Interfacial Tension Measurements

Interfacial tension (IFT) was measured via drop shape analysis using a drop shape analyzer (Krϋss Drop shape analyzer DSA 10, Hamburg, Germany). All measurements were made at 20 °C. The measuring cell was filled with isopropyl myristate (oil phase). In the next step, the water droplet was produced and allowed to equilibrate for 20 min, and at the same time, interfacial tension values were measured. An identical procedure was used for IFT measurements between oil and aqueous glycol solutions. The data shown are the mean value of three measurements.

### 2.7 Raman Spectroscopy and Confocal Microscopy

Raman spectroscopy was performed on a WITec α 300 spectrometer equipped with a confocal microscope. Measurements were made using a laser line with a wavelength of 532 nm, with a power of 30 mW. The laser was focused onto the sample through a 50× objective (NA = 0.75). The spectral resolution of the collected spectra was about 3 cm^–1^. Data analysis was performed using the TCA (true component analysis) technique. Raman maps reflect the distribution of a given component over the entire spectral range. Raman maps were recorded three times for each sample.

### 2.8 Differential Scanning Calorimetry (DSC)

Thermal analyses were conducted on a Mettler TA3000 calorimeter (Mettler Instrument, Switzerland) equipped with a TC 10 TA processor and a differential scanning calorimeter, using our previously reported procedures.^10^ Briefly, the microemulsion samples (15–30 mg) were weighted into 40 μL aluminum pans and immediately hermetically sealed. The samples were rapidly cooled by liquid nitrogen from ambient to −90 °C and then heated at a constant rate of 5 °C/min to 70 °C. An empty pan was used as the reference. The measurement results show different states of water in the tested microemulsions.

### 2.9 Stability during Storage

The microemulsion samples were stored in sealed glass vials at three temperature conditions: (i) *T* = 40 °C (elevated temperature); (ii) room temperature; and (iii) *T* = 5 °C (reduced temperature), for 14 days. In addition, the MEs were stored at room temperature for an additional 3 months. The selected conditions are the most frequently used in the stability assessment of cosmetic and pharmaceutical products.

### 2.10 Statistical Analysis

All of the results were expressed as the mean values ± standard deviation (*n* = 3). Student’s test was run to determine significant differences at a 5% significance level (*p* < 0.05).

## 3 Results and Discussion

### 3.1 Effect of Glycols on the Composition of Microemulsions

**Figure 1:**
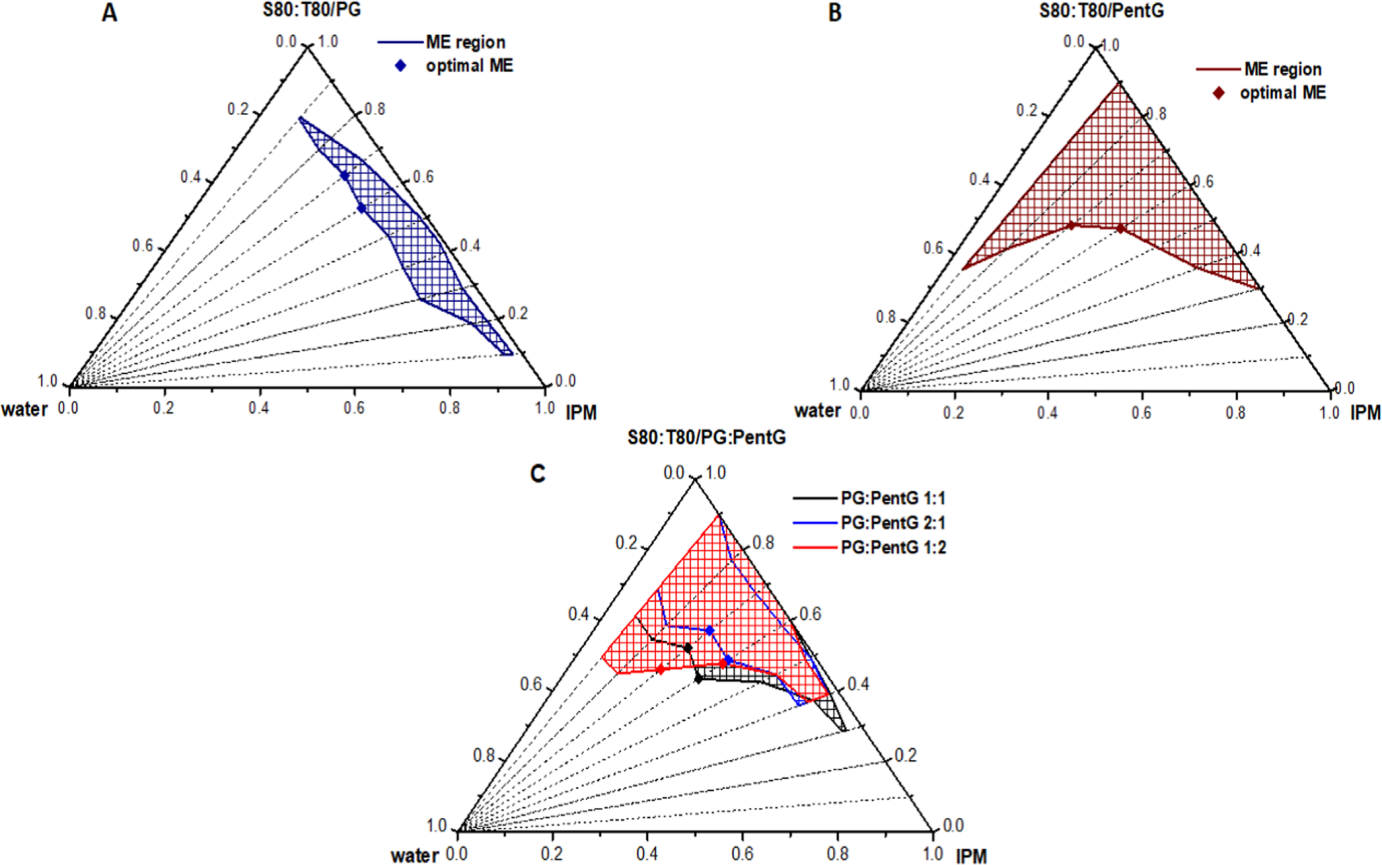
Phase diagrams showing the composition of MEs with (A) propylene glycol (PG), (B) pentylene glycol (PentG), and (C) mixture of propylene glycol and pentylene glycol in three different proportions. Optimal ME (marker) system selected for further evaluation.

At first, MEs with oil/surfactants/cosurfactant/water (IPM/S80:T80/glycol/water) were prepared. The areas of ME occurrence in the phase diagrams, depending on the glycol used, PG or PentG, are shown in Figure 1A,B. It can be seen that even the ratio of oil/surfactants + cosurfactant equal to 90:10 wt %, i.e., with a very high oil content, could be used to prepare ME with PG (Figure 1A). However, all MEs formed with PG contained relatively small amounts of water phase (less than 15%). On the other hand, MEs with the second tested glycol, PentG, were characterized by a much higher water content compared to the systems with PG (Figure 1B). It is well known that glycols, as cosurfactants, can incorporate into the surfactant layer at the interface and thus increase the interfacial fluidity.^11,13^ However, in the case of PG, part of its molecules will also decrease the polarity of water because PG is mainly soluble in water (log *P* = −0.9). In turn, PentG not only interacts with the surfactant layer but also is divided between the water and oil phases (log *P* = 0.2), which makes the interfacial film more flexible and increases the ME area in the phase diagram.

To identify possible synergistic interactions of PG and PentG, MEs with three different weight ratios of these glycols, i.e., PG:PentG 1:1, 2:1, and 1:2, were also prepared. The composition of these systems was plotted on the phase diagram, as shown in Figure 1C. The data showed that MEs with a mixture of PG and PentG may contain higher amounts of water compared to MEs with pure PG. However, the maximum amount of water that could be incorporated (44.7%) was smaller compared to ME with pure PentG (60.5%, Figure 1B). We also observed that as the amount of PentG in the glycol mixture increased, the ME area in the phase diagram increased. It can therefore be concluded that the content of both glycols significantly influences the range of usable concentrations of all components to obtain MEs.

From a technological point of view and in terms of cosmetic/pharmaceutical applications, a more acceptable ME composition is the one with a significant amount of water and oil phases and a reduced content of surfactants + cosurfactants. In our research, such a composition was shown by MEs with the ratio of oil/surfactants + cosurfactant equal to 30:70 and 40:60 wt % (marked as points in the diagrams). Therefore, only these two compositions were characterized in further studies. These MEs were called optimal and their quantitative and qualitative composition is presented in Table 1. Among the selected MEs, it can be seen that the systems with pure PentG and the systems with a higher content of PentG in glycols mixture (PG:PentG 1:2) have the most similar quantitative composition. This means that adding PG practically does not change the composition of this system. In addition, the assumption of this work was to produce MEs with a high content of water phase and a reduced content of surfactants, expressed as the water-to-surfactant ratio W/S > 1.^18^ As can be seen from Table 1, one system, i.e., with PG:PentG 1:2 fulfills this assumption. It can therefore be concluded that the extremely desirable composition of ME with a reduced content of surfactants can be obtained as a result of the correct selection of glycols.

**Table 1:** Composition and Structure of Optimal MEs^a^

	ME composition (%)						
glycols	oil	surfactants	cosurfactant	water	W/S	mean particle size (nm)	PDI
PG	26.67	41.48	20.74	11.11	0.27	151.2 ± 1. 8	0.277 ± 0.017
PG	35.09	35.09	17.54	12.28	0.35	136.8 ± 2.7	0.375 ± 0.047
PentG	20.69	32.18	16.09	31.04	0.96	82.9 ± 3.2	0.237 ± 0.006
PentG	31.62	31.62	15.81	20.95	0.66	104.9 ± 1.8	0.113 ± 0.009
PG: PentG 1:1	22.39	34.83	17.41	25.37	0.73	77.8 ± 0.9	0.286 ± 0.002
PG: PentG 1:1	29.85	29.85	14.93	25.37	0.85	86.4 ± 2.5	0.504 ± 0.103
PG: PentG 2:1	24.48	38.10	19.05	18.37	0.48	125.6 ± 1.3	0.245 ± 0.017
PG: PentG 2:1	32.52	32.52	16.26	18.71	0.58	111.5 ± 2.5	0.315 ± 0.068
PG: PentG 1:2	19.74	30.70	15.35	34.21	1.11	64.6 ± 2.1	0.521 ± 0.045
PG: PentG 1:2	31.87	31.87	15.94	20.32	0.64	127.6 ± 2.9	0.148 ± 0.022

a W/S, water/surfactants ratio.

The ME composition also influences the particle size of these systems. It was noticed that with the increase in the amount of the water phase in the ME, the diameter of the droplets decreased. The MEs with the smallest particles contained the highest amounts of PentG, so it was confirmed that PentG can increase the flexibility of the interface, which contributes to an increase in the water phase content and an increase in the stabilization and formation of smaller droplet size.

To confirm the different effects of glycols on the interface, the interfacial activity of glycol solutions was also examined in proportions identical to those used in MEs. Figure S3 (Supporting Information) shows that the glycols–isopropyl myristate systems had a lower interfacial tension as the amount of PentG in the systems increased. Therefore, it was shown that PentG is characterized by an appropriate log *P* but also reduces the interfacial tension more strongly, which facilitates the formation of ME, as compared to PG.

### 3.2 Structure of Microemulsions with Glycols

To identify the type and phase transitions of MEs during their formation while adding water, the electrical conductivity was measured using an aqueous solution of 10^–2^ M NaCl (instead of pure water). Figure 3A shows the results of these measurements obtained for two MEs, i.e., containing pure PG and a mixture of PG:PentG 1:2 w/w. The figures characterizing the changes in all other optimal MEs are shown in the supporting material (Figure S2). To find phase transitions, a derivative of conductivity (κ) with respect to water content (d(log κ)/d*W*) was calculated. As described previously,^13,19^ the water content at which the derivative reaches its maximum value is defined as the water percolation threshold. Below this water content value, the droplets in the w/o-type ME are separated from each other and are in a nonconductive oil phase (the system exhibits very low conductivity values). However, above a certain volume of the water phase in the system (the percolation threshold), the droplets begin to fuse and form clusters (aggregates). Under these conditions, i.e., during fusion and then redispersing, ions may “move” from one drop to another as a result of the “transient fusion–mass transfer–fission” process.^20,21^

In our study, only systems with pure PG did not change their structure and remained the w/o type in the whole range of water content (Figure 3A). On the other hand, in MEs with PentG, the association between droplets led to cluster formation, as the measurements indicated the occurrence of percolation threshold in these systems.

Interestingly, in MEs with a ratio of 30:70 wt %, the water content at the percolation point was dependent on the PentG content. We noted that, as the amount of PentG in MEs increased, the system percolation took place at a lower water content (the data presented in Figure S2). As we demonstrated earlier, PentG showed greater interfacial activity than PG (Figure S1). It can therefore be assumed that PentG in the water phase can decrease the interaction between the water molecules and the head groups of surfactants, leading to a weaker packing of the interfacial layer on the droplets and promoting the transfer of ions and water molecules between the droplets.^22^ In addition, Liu et al. noted that the water content in reverse micelles that induced percolation decreased with an increase in the molecular volume of the solvent.^23^ In the MEs we studied, some of the PentG molecules could be present in the oil phase, increasing its molecular volume. PG, on the other hand, is practically dissolved only in the water phase.

In MEs with a 40:60 wt % ratio, structural changes occurred at the water content of approx. 4 wt %, regardless of the PentG used. It can be assumed that in these systems, glycols did not affect the percolation value because most probably their contents in MEs were lower.

### 3.3 Spectral and Microscopic Characterization

**Figure 2:**
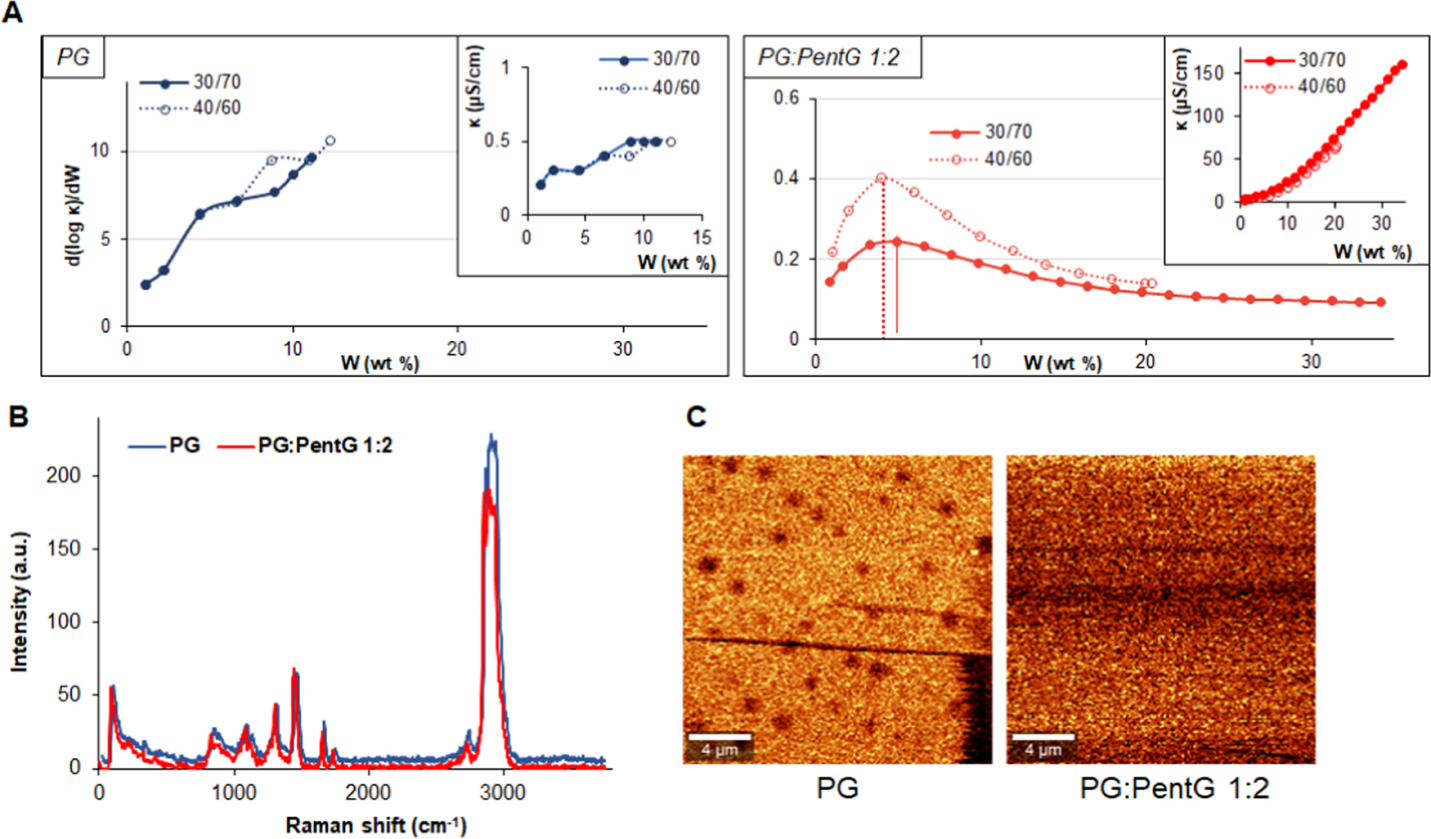
(A) Plot of conductivity and its log first derivative as a function of water content (*W*), (B) the Raman spectra (the intensity for the PG-based system has been shifted by 5 units so that the spectra did not overlap), and (C) confocal microscopy images; all figures correspond to microemulsions with pure propylene glycol (PG) and with a mixture of glycols (PG:PentG 1:2 w/w).

Figure 2B shows the Raman spectra for the same as above MEs, i.e., with pure PG and a mixture of PG:PentG 1:2 w/w. All main characteristic bands for these two systems are observed in an almost identical wavenumber range. The bands at 841 and 836 cm^–1^ are assigned to the hydroxyl end groups (OH), and the bands at 1085 and 1081 cm^–1^ are corresponding to the stretching mode of the C–O group in the polyoxyethylene chain of the surfactant (Tween 80) (for systems with PG and PG:PentG 1:2, respectively).^24^ The band observed at 1446 cm^–1^ is assigned to the aromatic ring of surfactants. The band at 1654 cm^–1^ may arise from very strong C=C stretching (unsaturated hydrophobic tail of surfactants).^25^ The bands observed at 2743 and 2736 cm^–1^ can be attributed to CH_2_, and bands in the range of 2850–2950 cm^–1^ are assigned to the aromatic C–H.^25^ However, no characteristic band for water was observed on the Raman spectrum, i.e., the O–H stretching, which produces a broad band in the range of 3100–3650 cm^–1^. However, it should be taken into account that water molecules in MEs can strongly interact with polar groups of surfactants and with glycols, especially with very hydrophilic PG. In such conditions, OH can form the strongest bonds, that is DAA-OH (DAA bond, single donor–double acceptor). This assignment is consistent with other studies,^26,27^ in which the OH vibrations associated with DAA ranged from 2950 to 3100 cm^–1^. Therefore, the band assigned to water was not visible on the Raman spectrum for our tested MEs in Figure 2B.

Confocal microscopy imaging of the above-described MEs (Figure 2C) confirms their structure, previously determined by electrical conductivity measurements (Figure 2A). The ME based on PG was characterized by separated spherical particles, indicating the w/o-type system. On the other hand, the ME with a mixture of glycols, i.e., PG:PentG 1:2, formed a cluster structure, similar to bicontinuous phases, without a clear division into the internal and external phases. It should be mentioned here that the particle size of the MEs tested was below the spatial resolution of the measuring device. Moreover, the recorded signal from a given area was averaged; therefore, the image did not reflect the actual particle size. This study was only used to determine the structure of the tested systems.

### 3.4 Rheological Properties of Microemulsions with Glycols

**Figure 3:**
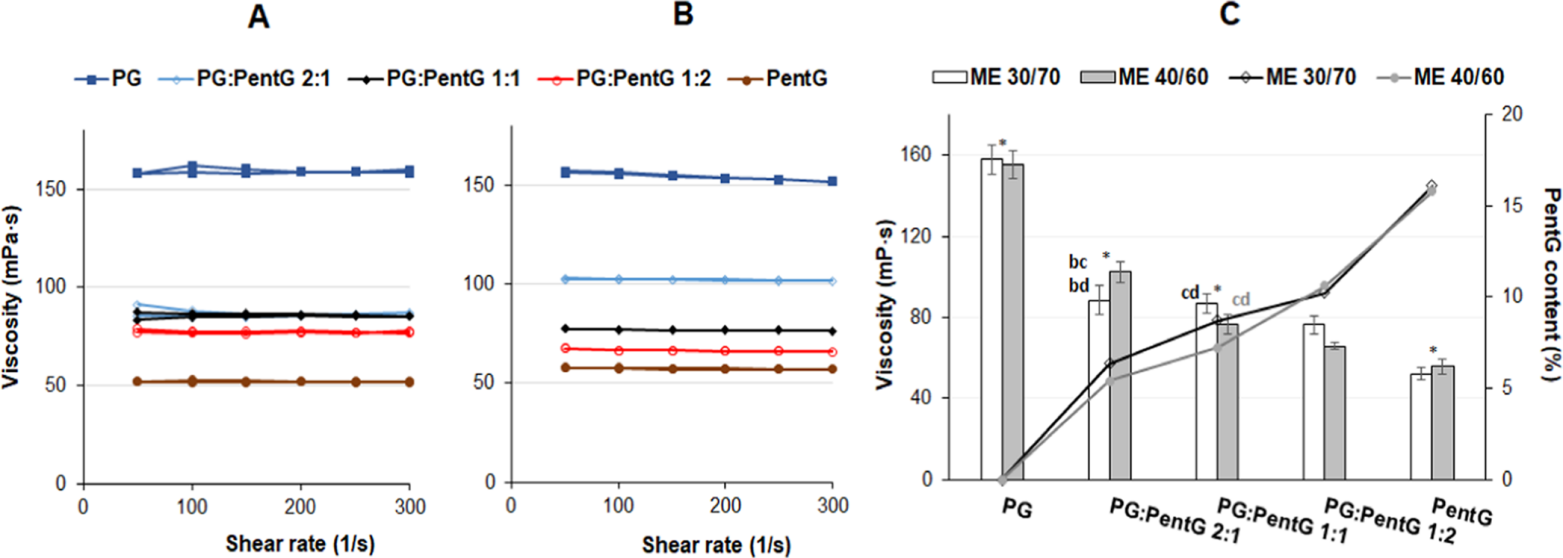
Rheological characteristics of microemulsions with different proportions of glycols. The weight ratios of oil/surfactants + cosurfactant were (A) 30:70 and (B) 40:60. (C) Viscosity as a function of the glycol content in microemulsions. The columns show the viscosity values. Means with * were not significantly different (*p* < 0.05; *n* = 3) between microemulsions with 30:70 and 40:60 wt % ratio. Different letters represent no significant difference (*p* < 0.05; *n* = 3) between systems with the same wt ratio but different glycols (black and gray letters for 30:70 and 40:60 wt %, respectively).

The viscosity of MEs was measured with increasing and decreasing shear rate (Figure 3). It was found that the viscosity of all MEs tested does not depend on the shear rate values. Therefore, MEs with PG, PentG, and a mixture of these glycols belong to the Newtonian fluids. Such rheological properties are characteristic of microemulsion systems and are consistent with previous literature data.^28^ It was noted that the viscosity values of the individual MEs did not change significantly with the oil/surfactant + cosurfactant ratio (30:70 or 40:60 wt %), except for the ME formed with the addition of PG:PentG 1:2 (Figure 3A–C). On the other hand, the proportion of both glycols in MEs had a great influence on the rheological properties.

It was found that with the higher content of PentG in MEs, the viscosity values of the systems were lower (Figure 3C). It is difficult to explain these results because the viscosities of both glycols were similar (our measurements indicated 56.4 and 62.3 mPa/s^–1^ for PG and PentG, respectively). There was also no simple correlation between the composition and the particle size that could explain the changes in the viscosity of the MEs.^29,30^ We can only assume that the structure of MEs influences their viscosity because MEs based on pure PG were characterized by the w/o type and showed a much higher viscosity (above 150 mPa/s^–1^) than other systems (about 50–100 mPa/s^–1^). In these remaining MEs with a mixture of glycols and with pure PentG, changes in the structure occurred due to the formation of clusters and microchannels. These changes could have resulted in lower shear resistance. As mentioned earlier, PentG affects the flexibility and fluidity of the interface, which could also lower the viscosity of the systems.

### 3.5 Thermal Analysis

**Figure 4:**
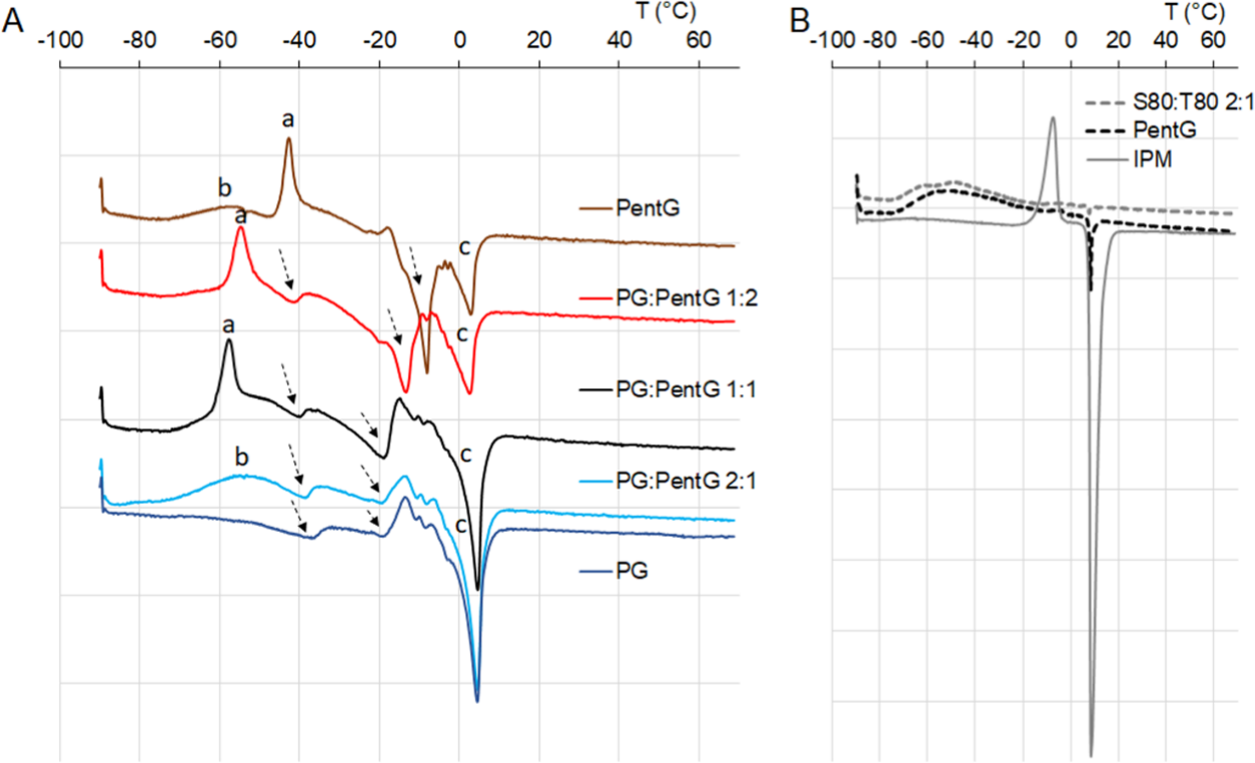
Thermal behavior of (A) microemulsions with different proportions of glycols (systems with the highest amount of water, see Table 1); (B) pure oil phase (IPM), pure pentylene glycol (PentG), and pure mixture of surfactants (S80:T80 2:1).

DSC measurement was performed for the MEs to determine different behaviors and states of water, depending on the ME structure and composition (Figure 4A). To identify all transformations on the ME thermograms, thermal analysis was also performed for pure components, i.e., oil, surfactant mixture, and PentG (Figure 4B).

The results of the DSC measurements may indicate water in three states, depending on the different mobility of the hydration layer around the polar heads of surfactants and cosurfactants: (1) loose or bulk water (freeze at approx. 0 °C), (2) interfacial**—**water loosely bound in the second hydration layer (melting temperature from −15 to −5 °C), and (3) bound**—**strongly bound water of the first hydration layer (melting temperature lower than −10 °C). Bound water molecules interact with surfactants so strongly that they cannot form hydrogen bonds with adjacent water molecules, which lowers the freezing point to very low temperatures. Nonfreezing water does not crystallize even at −100 °C.^10,31^

The DSC heating curves of the MEs with the addition of PentG show the exothermic peak (peak a), attributed to the cold crystallization of water (Figure 4A). It was reported that cold crystallization of water is the transition of amorphous ice to crystalline ice, observed in polymer–water systems (for example, polysaccharide–water, PMEA–water, or gelatin–water^31−34^). On heating, the water molecules solidified as amorphous ice. When the temperature rises above the glass transition, molecules can be mobilized and further heating enhances their molecular motion. As can be seen from Figure 4A, the cold crystallization was observed at about −40 °C in the ME with pure PentG, and the addition of PG to the ME systems resulted in a shift of this temperature to lower values (approx. −55 °C). The phenomenon of cold crystallization did not occur in systems with a higher PG content, i.e., PG:PentG 2:1 and with pure PG. Instead, peak b was noted and attributed to the crystallization of surfactants and PentG, as indicated by the thermal analysis of pure compounds in Figure 4B. The lack of cold crystallization of water may be due to the very strong interaction between water and PG, which is more hydrophilic than PentG. Also, the hydrophilic parts of the surfactants had weaker contact with the water molecules (PG steric effect, affecting the contact between surfactants and water). In addition, there was significantly less water in MEs containing a higher proportion of PG in the mixture of glycols.

The next water state, shown by arrows in the thermograms, relates to the interfacial water. For the ME containing only PentG, only one such peak was observed at about −10 °C. After the addition of PG, this peak shifted toward lower values (about −20 °C) and a second peak appeared at approx. −40 °C, indicating interfacial/bound water. Such a melting process of strongly and loosely bound water was also influenced by the strong interactions of PG–water, which lowers the melting point ranges for water.

The last peak *c* was related to the melting range of the oil phase as well as surfactants and PentG (above 0 °C). The above results indicate that depending on the composition, especially on the content of both tested glycols, MEs may show different thermal behavior. The presence of different water states in the delivery systems can in turn change the properties of such systems.

### 3.6 Stability during Storage

**Figure 5:**
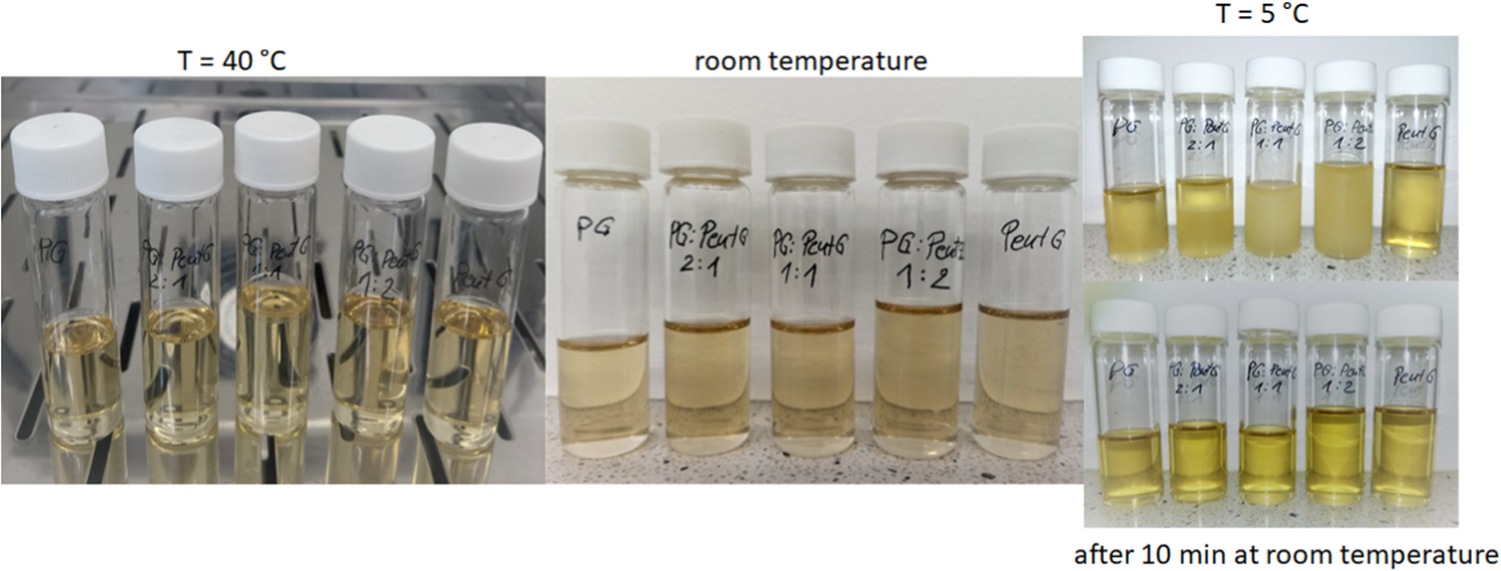
Appearance of the microemulsions with different proportions of glycols (PG and PentG) after 14 days of storage at elevated, room, and reduced temperatures (the bottom photo shows the samples taken out of the refrigerator and kept under room conditions for 10 min). The proportion of oil/surfactant + cosurfactant (O/S + C) in all samples was 30:70 wt %.

All MEs stored for 14 days under different temperature conditions (elevated, room, and cool) remained unchanged in appearance. No precipitation or phase separation was observed in the samples kept at 40 °C and room temperature. As can be seen in Figure 5, the samples were partially or completely cloudy upon removal from the refrigerator. However, after 10 min of keeping them at room temperature and gentle mixing, they became transparent and completely uniform again. Moreover, all MEs showed no change in appearance after 3 months of storage at room temperature.

### 3.7 Composition of Microemulsions with Selected Oils

MEs were also obtained with different oils, such as paraffin oil and medium-chain triacylglycerols (MCT), instead of IPM. The selected oils have a different structure (straight-chain paraffin oil, branched triacylglycerol), but each of them is safe and often used as a cosmetic and pharmaceutical ingredient.^35−38^ For this part of the research, three MEs were selected, i.e., with pure glycol:PG or PentG, and with one glycol mixture (PG:PentG 1:2), because in the above-described tests, they had the most desirable composition. The aim of this work was to determine the effect of the type of oil phase on the ME composition with selected glycols. Changing the hydrophilic–lipophilic interface, as a result of using a different oil, could have a positive effect on the lower content of surfactants necessary for the ME formation.

**Figure 6:**
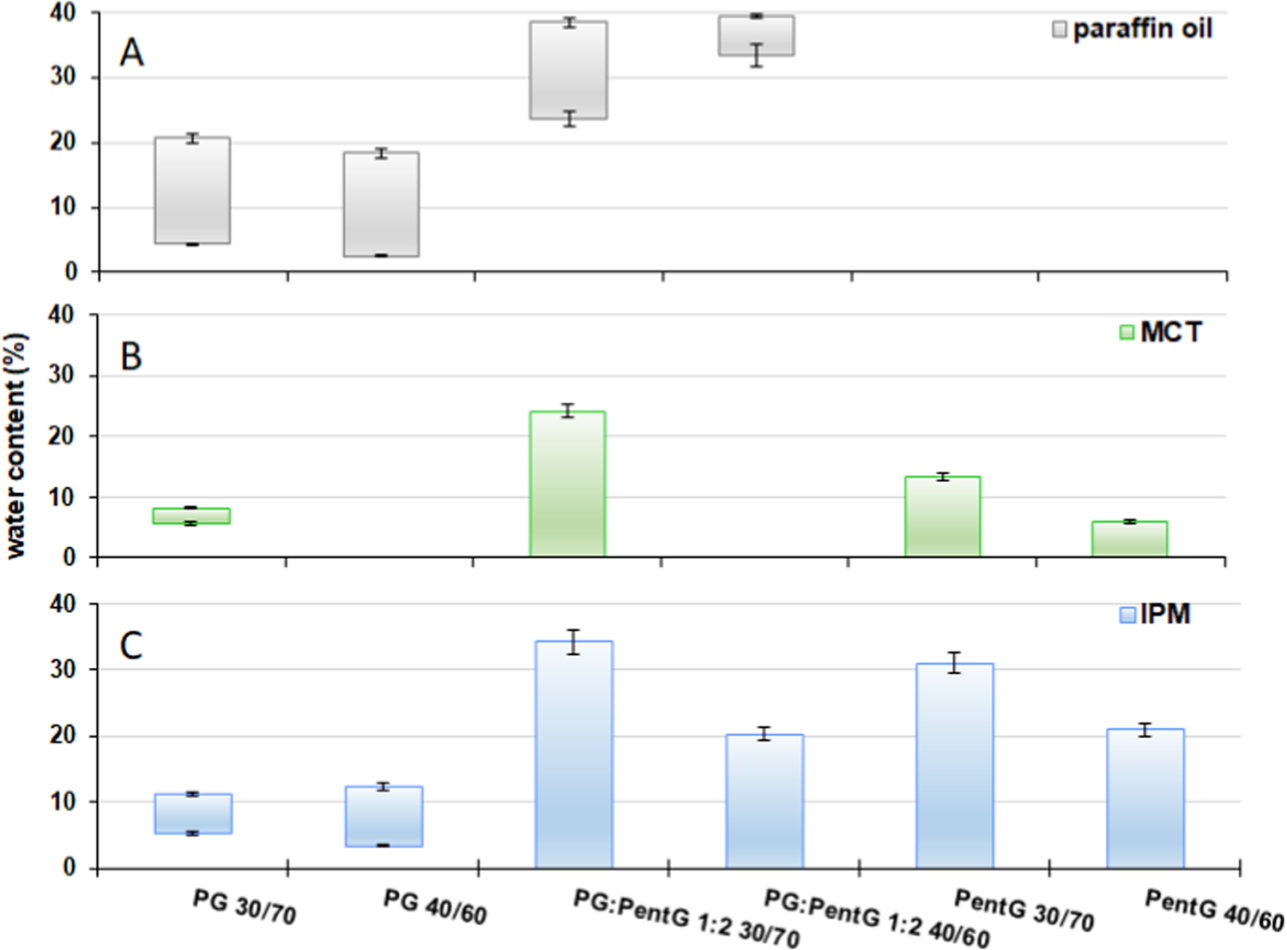
Water content range in MEs with selected oils: (A) paraffin oil; (B) MCT; and (C) IPM.

Figure 6A,B shows the ranges of the amounts of water in which the individual MEs were formed with the selected oils. For comparison, the previously discussed systems with IPM were also shown (Figure 6C). As can be seen, the type of oil phase and type of glycol affects not only the composition of MEs but also the possibility of obtaining MEs. If only PG was present in the system, the MEs contained the widest range of water (water quantity range in which a clear ME system was observed) after the use of paraffin oil. When the oil was changed to MCT, the water content in ME was very limited. System with 40 wt % of this oil did not even form ME. Looking at the structure and molecular weights of the tested oils (paraffin oil MW = 507 g/mol, MCT MW = 464.6 g/mol, IPM MW = 270.5 g/mol), it can be concluded that the formation of MEs with pure PG was more effective after using a high-molecular, straight-chain oil component, i.e., paraffin oil. It can be assumed that such an effect is related to the better arrangement of PG molecules at the interface in the presence of paraffin oil hydrocarbons. If the structure of the oil molecules was more branched, as in the cases of IPM and especially MCT, it could induce a specific hindrance to PG molecules at the interface and cause difficulties in forming and stabilizing the ME.

On the other hand, in systems with pure PentG, a greater range of ME formation was obtained when branched oil phases were used. The highest efficiency in ME formation was obtained after using IMP, probably due to the low molecular weight of this oil. In contrast, paraffin oil does not form a suitable composition with PentG to obtain ME. Similar results were published by Djekic and Primorac.^35^ The authors showed that MEs with higher water contents were formed in the presence of fatty acid esters with a low molar volume and relatively simple chemical structure. The use of oils with a high molar volume and complex structure (e.g., olive oil and mineral oil) did not promote the formation of MEs.

Interestingly, it was shown that the glycol mixture used to produce MEs showed synergism in the systems with paraffin oil and MCT. The amounts of water added to the MEs with MCT and PG:PentG were much higher as compared to the corresponding pure glycol systems. MEs with a mixture of glycols and paraffin oil were also obtained in a much higher range of water content as compared to MEs with pure PG. The high water content caused a reduction in the concentration of surfactants in these MEs, and as a consequence, the goal of water:surfactant ratio (W/S) above 1 was achieved (Table 2). It has therefore been proven that by appropriate selection of glycols and oil phases, the quantitative composition of the MEs can be significantly altered and adapted to the given application needs. However, these results require more extensive research to fully explain the possible mechanisms causing such effects. We plan to explore these issues in our next paper.

**Table 2:** Composition of MEs with Different Oil Phases

	ME composition (%)				
oil type	oil	surfactants	cosurfactants	water	W/S
paraffin oil	18.46	28.72	14.36	38.46	1.34
paraffin oil	24.24	24.24	12.12	39.39	1.63
MCT	22.73	35.35	17.68	24.24	0.69
IPM	19.74	30.70	15.35	34.21	1.11

## 4 Conclusions

In this paper, we presented the structure and compositions of MEs differing in cosurfactant. We used two glycols (PG and PentG) and their three mixtures with different proportions (PG:PentG 2:1; 1:1; and 1:2 wt %). The results indicate that the type, composition, and thermal and rheological properties of MEs can be manipulated by appropriate selection of the glycol composition. Despite the similarities in the structure of these compounds, belonging to the same group of chemical compounds (glycols), PG and PentG showed very different effects on the characteristics and properties of MEs. It has been shown that even the smallest addition of PentG induces a percolation threshold and changes in the structure of MEs and thermal states of water. Also, all MEs with the addition of PentG had a smaller particle size and higher water content compared to MEs with pure PG. These effects were attributed to the formation of a more flexible film by PentG molecules at the interface. In addition, it was initially shown that the tested glycols or their mixture showed different efficiency in the formation of MEs with oil phases of a specific structure. The correct selection of the oil phase and glycol composition also reduced the surfactant content in MEs. A more detailed understanding of this behavior is the next step to predict the composition and properties of MEs. Therefore, it is planned to expand this part of the research in the near future. However, the results presented in this paper provide important information on the selection of components needed to create and design MEs of a specific type and required parameters.

## Supporting Information Available

Interfacial activity of glycols; conductivity data as a function of water content for all microemulsions; and percolation threshold data in all microemulsions (PDF)

## Abbreviations

ME: microemulsion
PG: propylene glycol
PentG: pentylene glycol
S80: Span 80
T80: Tween 80
IPM: isopropyl myristate
MCT: medium-chain triacylglycerols
DSC: differential scanning calorimetry
W/S: water:surfactant ratio
