# Supplementary material for: Microemulsion Delivery Systems with Low Surfactant Concentrations: Optimization of Structure and Properties by Glycol Cosurfactants
Source: Mol Pharm. 2022 Nov 10;20(1):232–40. doi: 10.1021/acs.molpharmaceut.2c00599 (PMC9811459; doi:10.1021/acs.molpharmaceut.2c00599)
Supplement: Supplementary file 1 — mp2c00599_si_001.pdf [file mp2c00599_si_001.pdf]

**Microemulsion delivery systems with low surfactant concentration: optimization of the composition, structure and properties**

Patrycja Szumała[*a], Jolanta Kaplińska[a], Balbina Makurat-Kasprolewicz[ab], Szymon Mania[c]

[a] Department of Colloid and Lipid Science, Faculty of Chemistry, Gdańsk University of Technology, Narutowicza St. 11/12, 80-233 Gdańsk, Poland

[b] Faculty of Mechanical Engineering and Ship Technology, Gdansk University of Technology, Narutowicza St. 11/12, 80-233 Gdańsk, Poland

[c] Department of Chemistry, Technology and Biotechnology of Food, Gdansk University of Technology, Narutowicza St. 11/12, 80-233 Gdańsk, Poland

* Corresponding author. Tel.: +48 58 347 1523; fax: +48 58 348 6278; e-mail: patszuma@pg.edu.pl

Supporting Information:

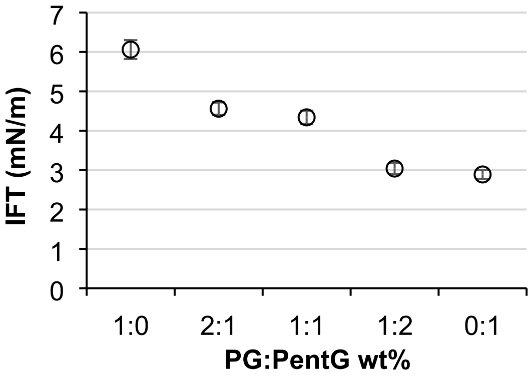


Fig. S1. Interfacial activity of glycols and their mixtures at the water-isopropyl myristate interface

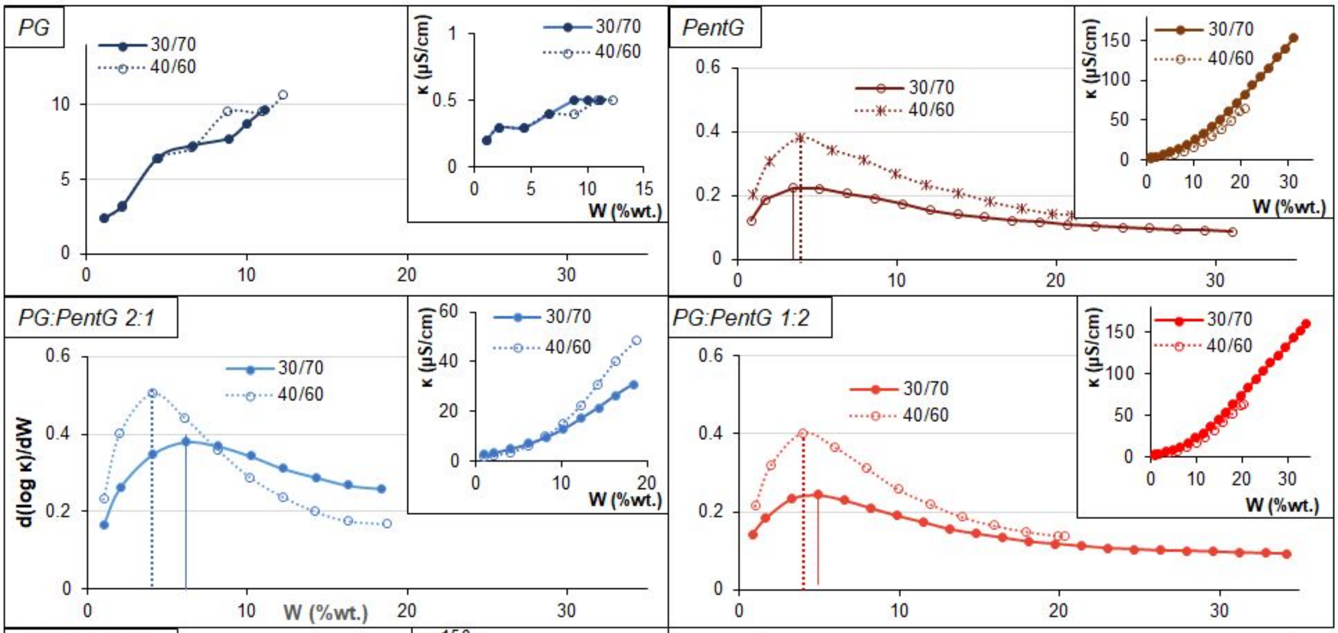


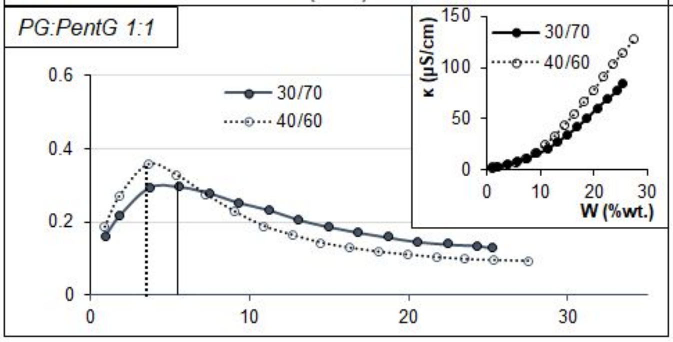


| Glycols | O/S+C (%wt.) | Water content at percolation threshold (%wt.) |
|---|---|---|
| PG : PentG 2:1 | 30/70 | 6.1 ± 0.2 |
| PG : PentG 2:1 | 40/60 | 4.0 ± 0.1 |
| PG : PentG 1:1 | 30/70 | 5.6 ± 0.2 |
| PG : PentG 1:1 | 40/60 | 3.9 ± 0.1 |
| PG : PentG 1:2 | 30/70 | 4.9 ± 0.1 |
| PG : PentG 1:2 | 40/60 | 4.0 ± 0.1 |
| PentG | 30/70 | 3.4 ± 0.1 |
| PentG | 40/60 | 3.9 ± 0.1 |

Fig. S2. Plot of conductivity and its log first derivative as function of water content (W) in MEs with glycols (PG and PentG) and theirs mixtures (2:1; 1:1; 1:2). Systems contained the oil/surfactant + cosurfactant (O/S+C) weight ratios 30/70 and 40/70. The data in the upper right corner indicate the percolation thresholds in MEs tested.
